# Galvanic Corrosion of Ti Dental Implants Coupled to CoCrMo Prosthetic Component

**DOI:** 10.1155/2021/1313343

**Published:** 2021-10-22

**Authors:** Francielly M. S. Soares, Carlos N. Elias, Emília S. Monteiro, Maria Elisa R. Coimbra, Ana Isabel C. Santana

**Affiliations:** ^1^Instituto Militar de Engenharia (IME), Material Science Department, Pr Gen Tiburcio 90, Rio de Janeiro 22290-270, Brazil; ^2^Universidade Estatual da Zona Oeste (UEZO), Av. Manuel Caldeira de Alvarenga 1203, Rio de Janeiro 23070-200, Brazil

## Abstract

Dental implant systems are composed of an implant, prosthetic components, and a crown. Since the implants are made of commercially pure Ti (cp Ti) and prosthetic components are often made of Ti and CoCrMo alloys, a galvanic couple between these two devices may lead to galvanic corrosion, ions release, and even loss of the implant. This study aimed to investigate the corrosion resistance and measure the galvanic potential between cp Ti alloys (annealed microstructured cp Ti G4 and cold-worked nanostructured cp Ti G4) and a CoCrMo alloy. The corrosion resistance has been characterized by measuring the open circuit potential, the potentiodynamic polarization, the potentiostatic polarization, and the zero-resistance current. The cp Ti has been tested before and after a surface acid treatment. The samples' surfaces have been examined by scanning electron microscopy, and their surface roughness has been measured by a 3D optical profilometer. The polarization results showed that the CoCrMo alloy showed lower corrosion resistance than cp Ti. The surface acid treatment improves dental implant corrosion resistance. The galvanic analysis showed that the cp Ti without surface treatment behaved as an anode and after the acid treatment has a cathodic behavior in relation to the CrCoMo alloy. The highest value of galvanic current was cp TiG4 acid etched in contact with CoCrMo, in pH 2 solution. The galvanic couple with the lowest current has been the nanostructured cp Ti in contact with CoCrMo alloy.

## 1. Introduction

**Figure 1:**
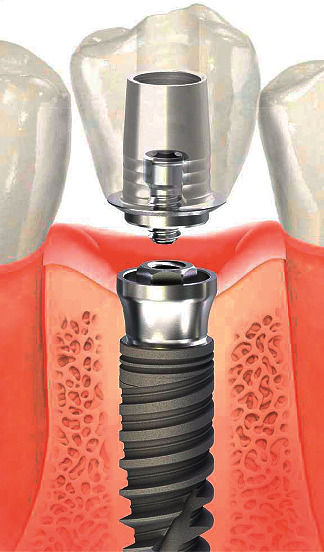
Design of a dental implant system: screw-shaped implant, a prosthetic component with screw, and crown. Courtesy of Conexão Sistema e Próteses.

A dental implant system consists of three main components: implant, prosthetic component, and crown. The implant is a cylinder, conical, or hybrid-shaped inserted into the alveolar bone for replacing the dental root (Figure 1). The prosthetic component is also named an abutment. The abutment is attached to the dental implant by a screw that raises it from the bone surface to above the gingival surface. The crown that replaces the visible part of the tooth can be either cemented or attached with a screw to the abutment [1].

Commercially pure titanium (cp Ti ASTM F67) is commonly used in dental implants due to a good combination of adequate mechanical properties with biocompatibility [2]. Favorable properties of cp Ti include adequate mechanical strength, chemical stability, good osseointegration, high strength-to-weight ratio, and corrosion resistance [3, 4]. Titanium has a strong affinity to oxygen, and a very thin and stable Ti oxide film has been formed spontaneously on its surface. This passive layer is responsible for osseointegration and offers good protection against corrosion or degradation [1, 5]. Oxygen ions present in the saliva may penetrate the oxide film and increase the oxidation of titanium, growing the thickness of the oxide layer [6].

The dental implant surface feature plays a very important role in the interaction with the proteins, glycoproteins, and cells. This interaction depends directly on some characteristics such as surface roughness, energy, wettability, and chemical composition. The rough and porous surfaces stimulate cell differentiation and the formation of extracellular matrix structures [7, 8].

Several techniques have been employed to change the dental implant surface morphology [1–3]. Titanium dental implant surface characteristics have been modified by acid etching, sandblasting, and anodizing. These surface treatments increase the surface roughness and area of implants, and the objective is to improve the cell interaction on the surface and corrosion resistance. The acid etching of titanium creates a surface micro- and nanoroughness that appears to enhance early dental implant osseointegration.

According to many studies into the oral environment, the presence of chloride and fluorine can change the corrosion resistance of metals [5, 8–11]. Furthermore, pH variations, bacterial plaque biofilm, temperature disturbances, food intake, and implant micromovement, including other variables, can also reduce the corrosion resistance of these alloys [5, 9, 10].

The release of ions in the human body leads to adverse health effects; besides, it reduces the durability of the materials. Large amounts of Cr ions released in the body can result in cancer and DNA damage. In addition, great quantities of Co induce cardiomyopathy, anorexia, and polycythemia diseases [5].

The contact through a physiological fluid of two or more medical devices manufactured from different metal alloys generates a potential difference, resulting in a flow of electrical current. In this case, it forms a galvanic cell. As a result of this galvanic current, the less noble metal has its process corrosive increased. In addition, this electrical current flows through the tissues, causing pain and local inflammation [9, 11, 12].

Abutments are connecting pieces that join the crown to the implants. They are manufactured with different metallic alloys, commonly with cp Ti, Ti-6Al-4V alloy, and CoCrMo alloy. When cp Ti dental implants are coupled with implant prosthesis structures made with cobalt-chromium alloys, nickel-chromium-titanium, gold-palladium alloy, and titanium alloy (Ti-6Al-4V) galvanic corrosion may occur [12].

The objective of this work was to investigate the corrosion resistance and galvanic corrosion of annealed microstructured commercially pure Ti grade 4 (Ti G4), cold-worked nanostructured commercially pure Ti G4 (Ti UFG), and CoCrMo alloy. This study is important due to the analysis of the influence of the oral environment on the galvanic corrosion and release of harmful ions of prosthetic structure supported by a dental implant.

## 2. Materials and Methods

### 2.1. Material

In the present work, Ti dental implants and prosthetic components alloys were used. The dental implants were made with annealed microstructured commercially pure Ti grade 4 (Ti G4) and cold-worked nanostructured Ti G4 (Ti UFG). The prosthetic components were made with CoCrMo alloy. Ti G4 unalloyed is specified by ASTM F67 technical standard. Ti UFG was produced by severe plastic cold deformation by equal-channel angular pressing (ECAP) at room temperature to refine grain size microstructure and increase mechanical properties [2]. The CoCrMo alloy is specified by the ASTM F1537 technical standard with a nominal chemical composition of Co-28Cr-6Mo. All specimens were supplied by Conexão Sistema de Prótese Co (Arujá, SP, Brazil). The sample surface acid treatment was the same as the available cp Ti dental implant. The samples were treated with an acid mixture (H_2_SO_4_ + HCl + H_2_O).

### 2.2. Surface Characterization

Specimens' surface morphology before and after acid etching was analyzed using the scanning electron microscopy (SEM) FEI Quanta FEG 250 (Field Emission Gun FEI Quanta FEG 250, Hillsboro, Oregon, USA).

Energy dispersive spectrometry (EDS) was used for the characterization of alloy chemical composition. The EDS analysis was made using a detector Bruker (Bruker Co, Durham-UK) for scanning electron microscopy.

Surface roughness was determined using a ZYGO 3D profiler model NewView 7100 (Zygo Co, Laurel Brook Road, Middlefield, CT 06455-USA)

### 2.3. Electrochemical Analysis

Electrochemical corrosion analyses were performed in the following electrolytes: NaCl 0.9% and a mouthwash solution commercially available (Plax®, Colgate Palmolive Industrial Ltda, São Paulo, Brazil). The mouthwash solution chemical composition is presented in Table 1. HCl was used for the pH adjustment of the NaCl solution. Two electrolytes pH values (2 and 6) were used. The objective of electrolytes with two pH is to analyze the influence of acid oral environments like orange juice and saliva. The literature result showed that the combination of low pH, and the presence of fluoride ions in solution severely affects the breakdown of the protective titanium oxide layer, leading to corrosion [13].

**Figure 2:**
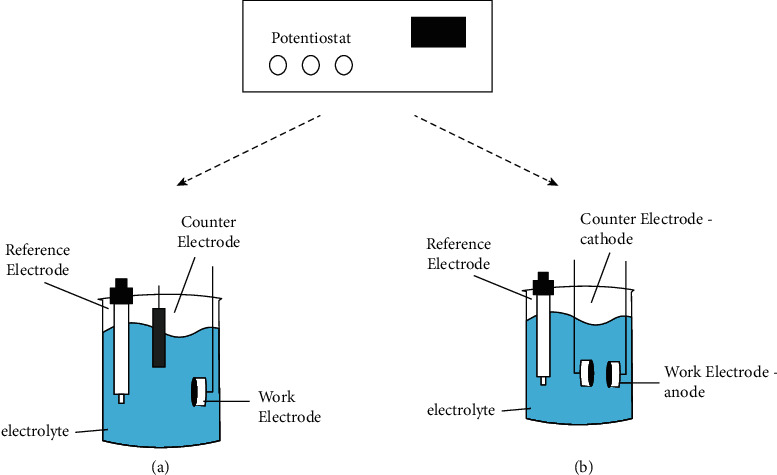
Schematic of (a) conventional corrosion cell and (b) galvanic electrochemical cell.

For conventional corrosion tests, a three-electrode electrochemical cell setup was used, which consisted of a saturated calomel reference electrode (SCE), platinum (Pt) counterelectrode, and a specimen (Ti or CrCoMo alloy) as the working electrode.  Figure 2(a) shows the cell setup of a test. For corrosion tests, the surface area of the working electrode was 0.2 cm^2^. The first corrosion analysis was to measure the OCP (open circuit potential). OCP is the metal equilibrium potential on the environment (electrolyte). The time to reach equilibrium depends on the metal alloy composition and surface finishing, and there is no technical standard specification to establish the testing time. For this study, after some preliminary testing, one hour was adopted. Electrochemical experiments were performed in the naturally aerated electrolyte.

Potentiostatic polarization is a fundamental technique to study the corrosion resistance of different alloys. The polarization curves were obtained after OCP measurements, with a scan rate of 0.01 V/s in the range from −1.0 V to 1.0 V.

Electrochemical analysis for OCP measurement and potential dynamic polarization curve was made with an Omnimetra Instruments, model PG-3901 potentiostat.

For galvanic measurements, the electrochemical cell was similar to the one used at electrochemical analyses, with a three-electrode cell setup used. Based on ABNT NBR 15613-5 (Implants for surgery: corrosion resistance) and ISO 10271: Dentistry-Corrosion test methods for metallic materials) technical standards, the galvanic current measurement was made using a counterelectrode as the cathode and the working electrode as an anode (Figure 2(b)). OCP measurement after 3,600 s stabilization was used as a parameter to determine the anode and cathode of current and galvanic potential analyses in different solutions.

Initially, for galvanic measurements, the CoCrMo alloy was used as anode and cp Ti G4 as the cathode, as expected by this galvanic couple. However, after the first measurement, a negative current (cathodic) was observed. The following analyses were made using cp Ti G4 as working electrode (anode) and CoCrMo alloy as the counterelectrode (cathode). The distance between anode and cathode was 0.5 cm. In this test, the reference electrode was saturated calomel (SCE), the working electrode was the anode, and the cathode was connected to the zero-resistance ammeter. The technique used was zero-resistance amperometry (ZRA). The galvanic corrosion experiment was carried out on an AUTOLAB potentiostat-galvanostat equipment model PGSTAT 302N.

Anode and cathode determination were obtained from OCP analysis for each material in each solution. The galvanic current was monitored for 24 h. All analyses were made in triplicate.

## 3. Results and Discussion

Ti surface morphology is an essential feature in dental implant osseointegration and rate treatment success. The osseointegration is influenced by the physical and chemical properties of the implant surface [14]. The surface morphology, energy, roughness, and type of oxide influence the interaction of the biomaterial with proteins and cells and the osseointegration mechanisms [15, 16]. Different types of dental implant surface treatments were developed to improve cell response and osseointegration [17, 18]. For example, acid etching, calcium phosphate blasting, Al_2_O_3_ blasting, Al_2_O_3_ blasting + surface acid treatment, TiO_2_ blasting + surface treatment with hydrofluoric acid, and anodizing were developed for dental implant osseointegration improvement [1]. But it is also important to analyze the corrosion resistance of the biomaterial.

The results of the specimen's chemical composition analysis obtained by EDS were close to those informed by the manufacturer and by the technical standard. The accuracy of the analysis of the chemical composition of the equipment was 99.73%.  Table 2 shows the chemical composition analysis results.

**Figure 3:**
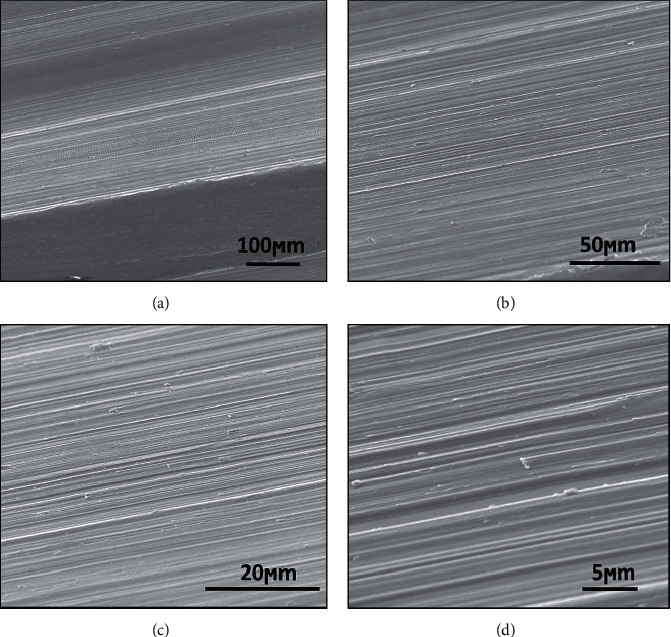
Annealed TiG4 surface morphology before acid treatment with different magnifications.

Figure 3 shows the surface of TiG4 without acid treatment. It is possible to observe the presence of grooves heritage from the machining process. Although titanium has osseointegration, the roughness surface is a very important factor. The morphology and roughness of the titanium implant surface have a great influence on osseointegration. Dental implants with low surface roughness (Ra = 0.5–0.8 *μ*m) do not have osseointegration and tend to form an encapsulation with fibrous tissue around the implant. Surfaces with high roughness (Ra > 2.0 *μ*m) have little osseointegration. Titanium surfaces with moderate roughness (Ra = 1.0–1.5 *μ*m) have better osseointegration and higher implant success rates [1, 14, 18].

**Figure 4:**
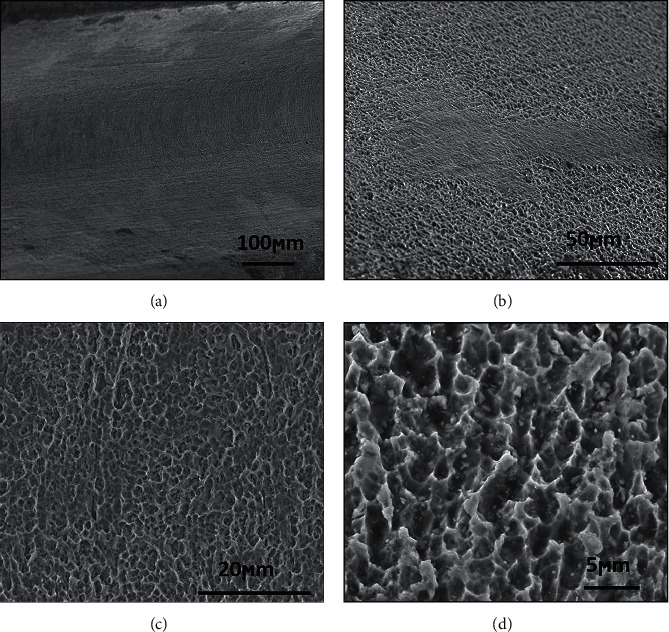
Annealed TiG4 surface morphology after acid treatment with different magnifications.

Figure 4 shows cp Ti G4 dental implant surface morphologies after acid treatment. The surfaces exhibited microcavities and roughness characteristics created by acid etching treatment. The microcavities and roughness on the surface increase the surface energy and roughness and could be decisive to protein and cellular interaction mechanisms. Microcavities with this size of the dental implant surface improve the protein-cell interaction and osseointegration mechanisms [1, 14].

Acid etching dental implant surface treatment can be done alone or after any blasting process, increasing the level and percentage of bone formation on the dental implant surface [14–18]. The objectives of dental implant surface acid treatment are to change the surface roughness, improve protein-surface interactions, and increase primary stability (mechanical), secondary stability (osseointegration), and tertiary stability (bone remodeling). The best dental implant surface roughness has Ra close to 1.0 to 1.5 *μ*m [1] like the morphology in Figure 4.

**Figure 5:**
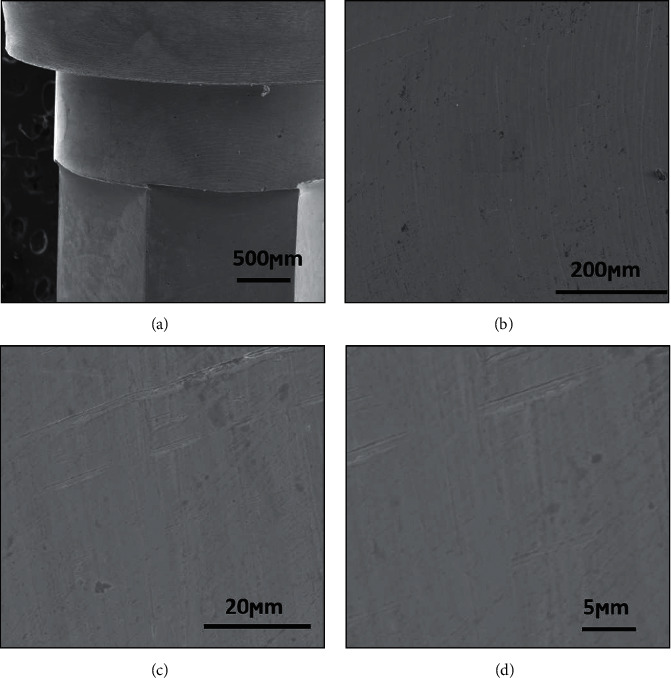
CoCrMo abutment surface morphology showing a smooth surface.

Figure 5 shows the surface morphology of the CoCrMo abutment. The morphology and roughness of the abutment surface are very different from the implant surface. The surface of the abutment is smooth, and the implant is rough. The roughness of the abutment is suitable for interacting with fibroblast-like cells and the implant surface to interact with osteoblasts. The commercial abutment did not receive acid treatment or mechanical surface treatment after machining, so its surface shows lower roughness than dental implants. The SEM images show a smooth surface with some grooves from the machining process.

Since the Ra roughness measurement parameter is more widely used in the dental implant literature, it was chosen to characterize the specimen morphology.  Table 3 shows the measured roughness parameters. The Ra value was different among specimens. The CoCrMo abutment showed the lowest Ra (0.28 ± 0.01 *μ*m) and cp TiG4 Ra (0.315 ± 0.09), and cp Ti G4 acid-treated showed the highest (1.42 ± 0.43 *μ*m). A significant statistical difference was observed between the TiG4 acid-treated and the abutment surface's roughness.

**Figure 6:**
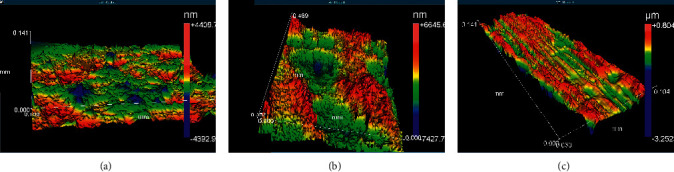
3D surface morphologies. (a) Abutment, (b) implant Ti G4 acid-treated, and (c) TiG4 without surface treatment.

Figure 6 shows that the abutment surface morphology was different from the surface of the dental implant. The CoCrMo prosthetic components were not submitted to acid surface treatment; they were only machined and had a smooth surface. The SEM micrographs of the components show that the component has lower roughness and porosity. Since no osseointegration occurs with the CoCrMo prosthetic components, there is no need to make any surface treatment. The smooth abutment surface would increase the fitting with the dental implant, improve fibroblast cell attachment, and decrease bacterial adhesion.

The surface roughness difference is directly related to the acid etching treatment processing. The morphology and roughness of the dental implant surfaces have a great influence on dental implant healing, osseointegration time, and the success rate of treatment [14]. Dental implants with Ra equal to 1.0–1.5 *μ*m surface roughness can improve cell interaction and primary mechanical stability and enhance osseointegration. Titanium dental implant with a rougher surface promotes a shorter healing process than smoother surfaces [19].

In the oral environment many factors, such as pH, the presence of chloride ions and fluoride ions influences corrosion resistance. So, a critical analysis of these factors concerning the corrosion resistance of the tested alloys was made. The evolution of OCP was measured at 3,600 s, and Table 4 shows the OCP values of annealed cp Ti G4 acid-treated, cp Ti UFG, and CoCrMo alloy. No significant corrosion potential behavior variation was observed among the alloys. The OCP values were approximately −150 mV. During the testing, there was great difficulty to identify the less noble material which can be used as anode or cathode in the galvanic analysis. The materials studied show a passive oxide film formation on the surface making them resistant to corrosion. Similar behavior was observed by Lu et al. [20].

**Figure 7:**
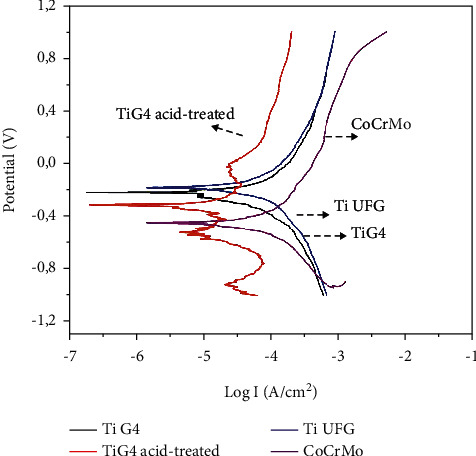
Anodic polarization curves of the alloys in NaCl 0.9% solution with pH 6.

Figure 7 shows the anodic polarization curve of cp Ti G4 after acid treatment, Ti UFG, and CoCrMo alloy in NaCl 0.9% solution with pH 6. Usually, the polarization curves with passive regions have three regions. In the first region, it is possible to determine *I*_corr_ (corrosion current) based on *E*_corr_ value (corrosion potential) and the transition of current from cathodic to anodic. The second region is related to the passive zone characterized by an oxide film formed on the surface of the material. The last region is the transpassive one, above the pitting potential, where the oxide film dissolution occurs. Some Ti alloys have not shown the transpassive region [12].

All analyzed metal alloys showed a passive region. The CoCrMo alloy showed a transpassive region (Figure 7). All *E*_corr_ values were very close, but cp TiG4 acid-treated had a displacement to the anodic direction. When the corrosion resistance of the as-received cp Ti G4 was compared to the Ti G4 acid-treated, it was observed that the acid-treated samples presented a lower current, which means higher corrosion resistance. The microstructured cp Ti G4 and nanostructured Ti UFG had the same chemical composition, but different grain sizes. This difference changed the corrosion resistance (Figure 7).

Samples of CoCrMo alloy had lower corrosion resistance than other alloys. This difference was observed mainly at the region of the anodic potential.

The analyzed alloys showed a passive region, except Co-Cr-Mo with a transpassive region (Figure 7 and Table 5). The *E*_corr_ of the alloys were very close, but cp Ti G4 acid-treated showed a displacement to the anodic direction. The irregularity that could be observed at the curves was due to the acid etching surface treatment. This kind of treatment increases the roughness and thickness of the titanium oxide film. When the corrosion resistance of the cp Ti G4 was compared to the cp Ti G4 acid-treated and Ti UFG, the last ones presented a lower current. This is directly linked to the different characteristics of oxide at the surface of the materials. These results indicate that the oxide film thickness, chemical composition, and grain size on the titanium surface influence corrosion resistance.

The measured electrochemical parameters are presented in Table 5. There is a difference among the samples' corrosion potentials (*E*_corr_). When the *E*_corr_ of the implant is compared with the *E*_corr_ of the abutment, a significant difference between them could be observed. Ti G4 acid-treated showed more positive values, but the passivation current (*I*_pass_) values were close to each other; the difference among them was 60 mA. Comparing the *I*_pass_ values of the dental implants with CoCrMo alloy, it was observed that they were lower.

For the potential and galvanic current measurements analysis, the galvanic couple among different alloys were determined. Anodic and cathodic choices for the couple were made based on the results of open circuit potential shown in Table 4. As the OCP results of each material were very close to each other, the choice was based on the alloy with the highest passivation.

**Figure 8:**
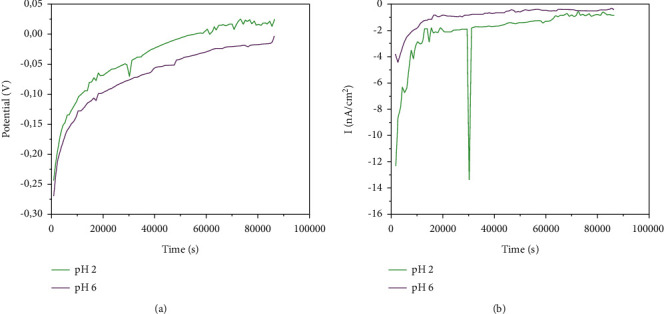
Galvanic potential curve (a) and galvanic current curve (b) of the couple CoCrMo – cp Ti G4 in NaCl 0.9% solution with pH 6 and pH 2. The CoCrMo alloy was the working electrode (anode).

Initially, CoCrMo alloy was used as an anode and cp Ti was a cathode. The results are presented in Figure 8.  Figure 8(a) shows that the galvanic potential started with negative values and became close to zero.  Figure 8(b) shows the galvanic current between Ti G4 and CoCrMo alloy. It is possible to observe that at the end of the galvanic corrosion testing the current of the couple got stable in negative values (cathodic) in both pH values (2 and 6) studied. This result indicates that CoCrMo alloy was not the anode but the cathode of the couple. The current generated was a cathodic one. As the solution got more acid, an increase of the galvanic current values was observed, showing that the pH of the solution influenced the material corrosion resistance and in the generation of the galvanic current.

**Figure 9:**
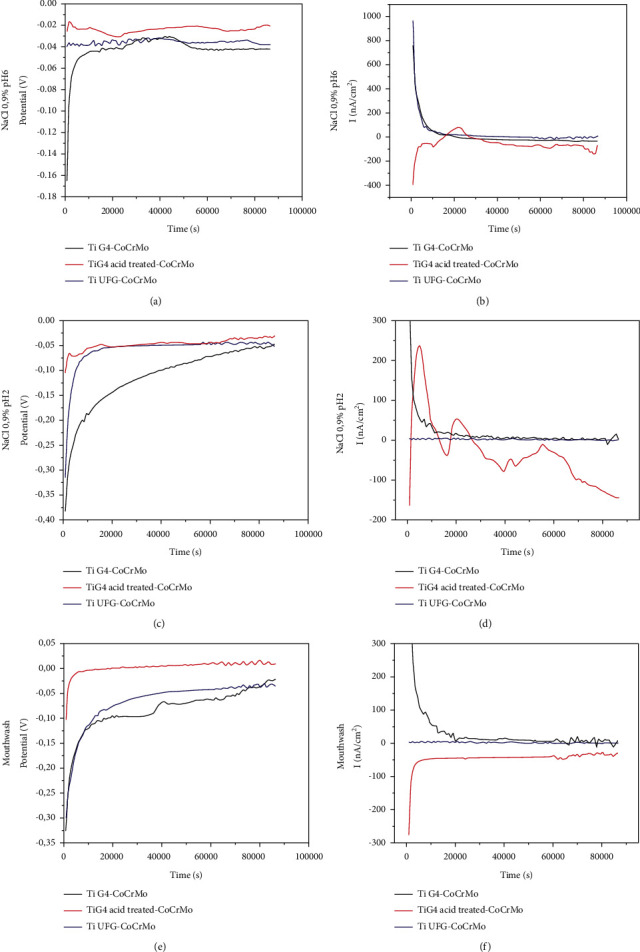
Galvanic potential curves and galvanic current curves of coupling cp Ti G4-CoCrMo, Ti UFG-CoCrMo, and cp Ti G4 acid-treated-CoCrMo. (a) and (b) NaCl 0.9% pH 6. (c) and (d) NaCl 0.9% pH 2. (e) and (f) Mouthwash solution.

After the first galvanic corrosion testing, it was observed that the titanium worked as an anode and not as a cathode as shown in Figure 8. Because of that, the electrodes were inverted, and CoCrMo alloy started to be used as the cathode and Ti as an anode of the galvanic couple. Figures 9(a) and 9(b) show the galvanic results of coupling cp Ti G4-CoCrMo, Ti UFG-CoCrMo, and cp Ti G4 acid-treated-CoCrMo. This analysis was made in NaCl 0.9% solution with pH 6. The galvanic potential measured during 24 h (86400 s) is shown in Figure 9(a). The potentials started with more negative values and raised to more positive ones, indicating that the film formation leads the potential to values close to zero. Before the analysis, the surface of all alloys was ground to remove the oxide film. All samples showed potential values close to zero. But the couple of cp Ti G4 acid-treated-CoCrMo was the one with values closer to zero.

Figure 9(b) shows the variation of galvanic current. Both couples with Ti G4 as anode showed more positive values up to a certain time. This behavior was also observed by Mellado-Valero et al. [12]. However, with the couple with cp Ti G4 acid-treated, the anode was CoCrMo. In this case, the Ti alloy chemically acid-treated had greater corrosion resistance and was the cathode of the galvanic couple. The acid etching surface treatment of cp Ti G4 affects directly the formation of the galvanic couple, cell potential, chemical reaction, and current between the dental implant (cp Ti G4) and abutment (CoCrMo). This result shows that the advantages of Ti dental implant surface acid treatment are that it improves osseointegration and increases corrosion resistance in the oral environment.

Figures 9(c) and 9(d) show the galvanic potential among the couple's specimens in NaCl 0.9% with pH 2. The initial potential of the couple of cp Ti G4-CoCrMo and Ti UFG-CoCrMo started in a more negative value and coming close to zero during the analysis. However, the couple of cp Ti G4-CoCrMo showed that the final potential is more negative than the other samples studied. The couple of cp Ti G4 acid-treated-CoCrMo showed that initial current values are more positive, and then, these values got closer to zero. The same was observed with the couple of Ti UFG-CoCrMo. This behavior is directly related to a passive quality film on the surface of the materials because the galvanic potential became almost null, which can avoid galvanic corrosion reactions.

The galvanic current observed in the corrosion testing of the couple of cp Ti G4 acid-treated-CoCrMo started with negative values, became positive, and then returned to negative values. This result shows that pH variation affects the kinetics of the reaction between the materials. The cp Ti G4 acid-treated can behave as an anode during some time and as a cathode during others.

Mouthwash solution is worldwide used to prevent bacterial plaque and as a coadjutant in oral hygiene. The prescription indicates the use twice daily. However, some care must be taken for the continuous use of mouthwash. Most people think that it can be used many times a day, and there is no damage to health. The use of mouthwash that was not prescribed could cause cancer and yellowing of the teeth and decrease the taste sensitivity. Another effect is on the dental implant surface because the fluoride affects the corrosion resistance.

Figures 9(e) and 9(f) show the results of the galvanic potential on the coupling in a mouthwash solution. The behavior of the Ti alloys is similar in the NaCl electrolyte. The potential started negative and coming close to zero as time elapsed. The cp Ti G4 acid-treated-CoCrMo couple was the one that was closer to zero. The galvanic current observed presented similar behavior to the curves obtained in the analysis aforementioned. The alloys previously ground (cp Ti G4 and Ti UFG) showed current with positive values at the initial hours and decreased as time elapsed. This behavior was observed in almost all the solutions analyzed. For the cp Ti G4 acid-treated specimen test, the behavior was different. Initially, the current was negative, and after some time, it came close to zero. The current stayed negative during all tests.

The current of the galvanic of dissimilar alloys placed in direct contact within the oral environment shows very low value, just around ten nanoamperes for most of the studied couples. Reclaru et al. [21] define significant galvanic currents above 15 mA/cm^2^. However, more detailed studies did not show an acceptable level of galvanic currents for biomaterial applications. Among the materials judged in this study, only one condition (a galvanic couple of cp Ti G4-CoCrMo in 0.9% NaCl solution and pH 2) showed galvanic current values close to the suggested as significant by Reclaru et al. [21].

Taher and Jabab [22] report that the chemical composition of the alloy, porosity, and polishing of the sample influence the corrosion resistance and affect the galvanic behavior of the materials. In keeping with these aforementioned results, it is possible to understand why titanium has had two distinct behaviors. In one condition, it works as an anode and in another as a cathode. Once that the oxide film on the surface of acid-etched titanium is more stable, it can increase the corrosion resistance of the acid-treated surface in comparison with the polished one [23]. Thus, in the galvanic pair formed with the acid-treated alloy, the titanium presents cathode behavior (more noble behavior). In samples without acid etching surface treatment, the titanium works as the anode, i.e., lower noble behavior, with limited corrosion resistance. One can argue that the surface treatment is an outstanding requirement in the corrosion resistance of titanium alloys, above all in circumstances where a galvanic pair can be formed. This result shows that special care should be taken when using cp Ti and titanium alloy implants without surface treatment. An example is Ti-6Al-4V total hip prostheses that are in contact with other metallic alloys. The results of the corrosion tests showed that galvanic corrosion increases. Mellaro-Valero et al. [12] observed similar behavior when they studied the cp TiG2 galvanic pair coupled to the CoCrMo alloy. In their results, they could observe that cp TiG2 showed a preferential dissolution and the observed current values were negative. Although the studied alloys exhibit high corrosion resistance, it is important to emphasize that it is fundamental to analyze their behavior when they are coupled with different materials.

Table 6 presents the galvanic potential and galvanic current values obtained after 24 h in the corrosion test. Initially, as observed using CoCrMo as the anode, the values of galvanic current were negative. This result indicates that Ti G4 was the anode of the reaction. When CoCrMo was used as the cathode and Ti G4 was used as the anode, the current turned anodic, except for Ti G4 acid-treated in NaCl 0.9%, pH 2 solution. These results showed that the surface treatment affects the corrosion resistance of Ti alloy.

The intensity of galvanic current was higher for a couple of Ti G4 acid-treated/CoCrMo. The acidification of the solution CoCrMo oxidation was faster, showing that pH influenced directly the galvanic corrosion of this couple. For all other coupled alloys, the corrosion current was insignificant. CrCoMo alloys showed good corrosion resistance in 0.9% NaCl solution at different pH values (2, 3, and 6) and mouthwash solution. But the corrosion current, particularly in cathodic potentials, increases as the pH decreases [24].

The values of galvanic potential after 24 h for all coupling were very close to zero due to the Ti oxide film on the surface. The results of the present work show that cp Ti alloys have different corrosion behaviors before and after surface acid etching treatment. The longer the surface is exposed to the acid, the greater the roughness and the acid surface attack, and the greater the thickness of the titanium oxide film is.

## 4. Conclusion

It can be concluded from the corrosion tests that all the studied alloys have had high corrosion resistance, and all of them form a passive oxide film. The polarization result tests showed that the CoCrMo alloy has lower corrosion resistance than titanium alloys. The Ti acid surface treatment improved its corrosion resistance. The galvanic analysis results showed that the Ti alloys without surface treatment behaved as an anode in contact with the CoCrMo alloy and after the acid treatment surface behaved as a cathode.

## Figures and Tables

**Table 1:** Mouthwash chemical composition (Plax®, Colgate Palmolive Industrial Ltd., São Paulo, Brazil).

Cetylpyridinium chloride (%)	Water	Glycerin	Potassium sorbate	Sodium fluoride (ppm)
0.075	—	—	—	225

**Table 2:** Semiquantitative chemical composition of analyzed alloys using EDS.

	Ti G4		Ti UFG		CoCrMo				
Element	Ti	C	Ti	C	Co	Cr	Mo	O	C
% weight	99.32	0.68	96.96	3.04	68.75	27.05	3.76	0.22	0.22
% atomic	97.34	2.66	88.90	11.10	66.35	29.60	2.23	0.80	1.02
Error 3 sigma (%weight)	0.09	0.03	1.32	0.33	1.78	0.69	0.15	0.08	0.10

**Table 3:** Roughness parameters, Ra (medium roughness).

Alloys	Ra (*μ*m)
CoCrMo abutments	0.28 ± 0.01
TiG4	0.315 ± 0.09
Ti G4 acid-treated	1.42 ± 0.43

**Table 4:** Measured open circuit potential (OCP) of the materials in NaCl 0.9% solutions after 3,600 s and pH 6.

Alloys	OCP (V)
cp Ti G4	−0.1500
cp Ti G4 acid-treated	−0.1503
cp Ti UFG	−0.1503
CrCoMo	−0.1498

**Table 5:** Electrochemical parameter measured by anodic polarization.

Materials	Solution	*E*_corr_ (v)	*I*_pass_ (A/cm^2^)
As-received cp Ti G4	NaCl 0.9%-pH 6	−0.2096 ± 0.020	2.69 × 10^−4^ ± 0.362
cp Ti G4 acid-treated		−0.3149 ± 0.131	1.25 × 10^−4^ ± 0.192
cp Ti UFG acid-treated		−0.2115 ± 0.038	4.16 × 10^−4^ ± 0.372
CoCrMo		−0.4427 ± 0.009	6.31 × 10^−4^ ± 0.350

**Table 6:** Galvanic current (*I*_Galv_) and galvanic potential (*E*_Galv_) in electrolytic with different electrolyte pH values after 24 h.

Specimen	Electrolyte	*I*_Galv_ (nA/cm^2^)	*E*_Galv_ (V)
CoCrMo-Ti G4 (cathode)	NaCl 0.9%-pH 2	−8.54	0.023
Ti G4 (anode)-CoCrMo		35.10	−0.042
Ti G4 acid-treated-CoCrMo		−144.42	0.010
Ti UFG-CoCrMo		7.32	−0.038
CoCrMo-Ti G4 (cathode)	NaCl 0.9%-pH 6	−4.15	−0.003
Ti G4 (anode)-CoCrMo		2.29	−0.051
Ti G4 acid-treated-CoCrMo		−71.26	−0.030
Ti UFG-CoCrMo		1.04	−0.047
Ti G4-CoCrMo	Mouthwash	7.32	−0.020
Ti G4 acid-treated-CoCrMo		−29.69	−0.021
Ti UFG-CoCrMo		1.04	−0.035

## Data Availability

The corrosion data and all analyses used to support the findings of this study are included within the article.
